# Chicago Dental Society

**Published:** 1885-03

**Authors:** A. E. Baldwin


### CHICAGO DENTAL SOCIETY.

### FEBRUARY MEETING.

REPORTED BY A. E. BALDWIN, M. D.

The paper of the evening was read by Dr. J. H. Wooley. His subject was "The First Permanent Molar." He premised by saying that the greatest tendency of professional men is to hobbies and narrow views. Opinions should be the result of careful research and study. The subject of the paper gives rise to many opinions. In the brief time allotted to me, deserved attention cannot be given. I can hope to bring out but few of the many points. To successfully elaborate this subject requires much study and long observation.

The jaws and teeth of children are formative material, to be modeled by us. To get good results requires perseverance and toil. Mistakes are often made by us all. I would compare our subject to a waif in the street, ignorantly neglected as to its value. The condition of the teeth is from heredity, pre-natal influences, combined with condition of the child before and after eruption. At the time of its eruption physical causes may disturb its normality, as in systemic disease; also that the tendency of the age is to develop in the child the highest type of nervous hyperæsthesia. The brain develops most rapidly at this time, and at the expense of the physical body; even the bones and teeth suffer from this cause. These facts, aided by the brain-forcing the child receives, show plainly why diseases of childhood retard physical as well as normal tooth development.

This tooth is often ignorantly neglected by parents, supposing it one of the temporary set; they are erupted at a time when often many of the temporary set are decayed, and the mouth in a condition to advance decay. If parents knew of the time of its eruption, and would give their children proper dental care, much less trouble would be experienced.

This tooth, according to Dr. Tomes' record of three thousand extractions, was removed in at least one-third of the cases. I hardly consider this a fair test, as a majority of these occurred in hospital practice. Many believe them to be very short-lived, from a consideration of the circumstances of their eruption, but think they should be preserved till the appearance of the twelfth year molar. Many also think them more liable to decay, claiming the time of eruption and condition of the mouth at the time, as proof.

Some claim that by removal of these teeth space is gained for correcting irregularities and preventing decay in adjoining teeth.

In my opinion the tooth, appearing as it does while many of the temporary teeth are still present, added to the carelessness of parents, renders the loss unnoticeable. Usually the dentist only sees the patient when acute pain is experienced in the tooth. Perhaps the decay is of slight extent, and easily filled. Often, owing to restlessness of patient, the work is hurried and necessarily slighted, resulting in failures. Dentists often think themselves irresponsible in case of failure, at least to the patient. This is very wrong. I think his work should be done with great care, and the parent should be told of the importance of attention to these, as to other teeth. Many satisfy themselves by thinking that by extraction of these teeth the second molar will move forward, and the bicuspid teeth backward, closing the space, and thus saving from proximal decay. I believe this occurs (*i. e.*, preventing decay) rarely, and that the law of the movement of teeth is that they are prone to a *forward* movement, only very exceptionally moving the opposite. Dr. Arthur says that in these cases of extractions for crowded arches, mechanical means must be resorted to, and cites a case where, following extraction, the second molar moved forward, closing the space entire, and that in this case proximal cavities were formed in all the teeth. I do not think they should ever be extracted for correcting irregularities, and I would question the statement that irregularities are due to lack of room. Dr. Kingsley thinks, outside of heredity and mechanical causes, that lesions or innervation of the trigeminus nerve cause these irregularities; that it is interference more or less prolonged, operating centrally with one of the prominent functions of that nerve, He admits that this is very difficult to prove, save by secondary phenomena, which must originate from such a source. As we all know, this nerve governs the supply of nutrient material to the parts it supplies. He (Dr. K.) farther thinks that at formative and eruptive stages of permanent teeth they are supplied with an independent vital or nerve force, which builds them up regardless of the slower development of the adjacent parts, thus crowding them into the undeveloped maxilla, and they forever retain this disordered arrangement in which the formation of the crown was formed, and they remain in a fixed position, regardless of subsequent growth of the jaw; for he says that it is one of nature's laws that at complete development function ceases.

Dr. Tomes also says that the teeth are not placed in a bony position, ready-formed; on the contrary, the bone surroundings, primarily, are absorbed, and when in position new bone is formed about them. He also says the size of the dental arch is not fixed by the size of the jaw. This last statement of Dr. T. has been confirmed by others, and in my own experience, in a great majority of my cases of irregularity, I find plenty of room in the jaw to arrange teeth without extracting our first molars. Dr. Lord believes they should not be extracted, and agrees with Dr. Arthur, that these are the most important teeth in preserving the size and shape of the jaw, and preserving the teeth in natural strength and position, and that often after their extraction we see such changes in the position of second molar and bicuspids as to interfere with normal mastication, and thus injurious force is brought upon front teeth. He thinks attention to them will prevent decay. With small cavities treat the same as any other tooth; with badly decayed teeth, break down to the solid borders, and fill, but do not restore.

I believe that in the extraction of these teeth a change is made in the expression of the face. In regulation we have to consider facial expression, and the effect of the shape of the arch on the voice. The wider the arch the fuller and rounder the tone. Dr. Black says that one great point of usefulness of this tooth is that it keeps the jaws in their relative position during the process of second dentition. He thinks this a very important fact—a fact noticeable when lost prior to eruption of the twelfth year molar, and thinks that if kept till the child is over twelve the chances are good for permanent retention. He farther says his study of the comparative liability of decay occurring at different periods is, that these teeth are attacked oftener for two or three years after eruption, and after that less often; and that if first and second molars are normal at fifteen, the chances are better for the first than the second. Posterior proximal decay occurs on second much oftener than on the first molars.

Now, I do not lay the blame of all mal-positions to the foregoing causes, for there are other reasons. Some claim that this tooth is frail. It is my experience, in many years of observation and treatment, that if as zealously cared for as others they can be saved as well. Should always recommend the use of the dam in thorough examinations of these teeth, often being enabled by so doing to locate slight decalcification, and to fill with small labor what, if neglected, would rapidly ruin the whole tooth.

In regard to mesial surfaces, if the temporary molar is in close contact cut away its distal surface, and if the mesial surface is superficially decayed remove and polish. Whether at any time you extract any or all of these teeth, it is rare that existing evils are removed.

Now, a few words in regard to treatment of these teeth. I find that experience aids greatly, and I have learned that it is erroneous to treat as in other teeth. They require greater care. I now, in large cavities, sensitive, without pulp exposure, fill with one of the cements, and let it remain months; first in filling, flowing the cement over the bottom of the cavity, then, as it hardens, finish with the same mixed harder. When the filling begins to wear, I remove around the margins and surface and fill with material best adapted. I think these teeth more sensitive to thermal changes than any other teeth. I believe also (and would emphasize this), that the pulps of these teeth are more sensitive for the first few years after eruption than any of the other teeth. When these teeth, from eruption, are cared for properly, and their nature and resources are well understood, the admitted verdict will be that it will be the fault of the dentist, and not of the teeth, if they are not saved.

(NOTE.—In the discussion of this paper by Drs. Allport, Crouse, Nichols, Matteson, Ames, Noyes, and others, many very excellent points were brought out; but, depending upon notes taken at the time, which I have unfortunately lost, I am unable to report the discussion intelligently. A. E. B.)

The next meeting occurs March 3d, 1885, when Dr. Harlan reads a paper. Subject: "The Antrum Highmorianum; Diseases and Treatment."

